# Ultrasonographic assessment of lower limb muscle architecture in children with early-stage Duchenne muscular dystrophy

**DOI:** 10.1590/0004-282X-ANP-2021-0038

**Published:** 2022-02-21

**Authors:** Numan BULUT, Ayşe KARADUMAN, İpek ALEMDAROĞLU-GÜRBÜZ, Öznur YILMAZ, Haluk TOPALOĞLU, Levent ÖZÇAKAR

**Affiliations:** 1 Hacettepe University, Faculty of Physical Therapy and Rehabilitation, Ankara, Turkey.; 2 Lokman Hekim University, Faculty of Health Sciences, Department of Physiotherapy and Rehabilitation, Ankara, Turkey.; 3 Hacettepe University, Medical School, Department of Pediatrics, Division of Pediatric Neurology, Ankara, Turkey.; 4 Yeditepe University Hospital, Department of Child Health and Diseases, İstanbul, Turkey.; 5 Hacettepe University, Medical School, Department of Physical Medicine and Rehabilitation, Ankara, Turkey.

**Keywords:** Muscles, Ultrasonography, Muscular Dystrophies, Architecture, Physical Functional Performance, Músculos, Ultrassonografia, Distrofias Musculares, Arquitetura, Desempenho Físico Funcional

## Abstract

**Background:**

Muscle imaging methods such as ultrasound and magnetic resonance imaging have been used for many years to determine the dystrophic process in muscular dystrophies. However, the knowledge regarding muscle architecture in children at early-stage Duchenne muscular dystrophy (DMD) with different functional levels is limited.

**Objective:**

To explore the effect of functional level on muscle architectural properties in children with early stage DMD and the difference between DMD and typically developing (TD) peers.

**Methods:**

Thirty children with DMD (15 Grade 1 and 15 Grade 2 according to the Vignos Scale) and 5 TD peers were included. Ultrasound imaging was used to measure muscle thickness (MT), fascicle length (FL), and pennation angle (PA) of vastus lateralis (VL) and medial gastrocnemius (MG) muscles bilaterally.

**Results:**

The MT and FL values for VL, and MT, FL and PA values for MG muscles were higher in children with DMD compared with those of TD peers (p<0.05). The FL of VL, and MT and FL of GM muscles of children with DMD Grade 2 were higher than those of children with DMD Grade 1 (p<0.05).

**Conclusions:**

MT and FL are increased in children with DMD compared with TD peers. Additionally, muscle architecture seems to be affected even at the early stages of the disease.

## RESUMO

**Antecedentes:**

Métodos de imagem muscular, como ultrassom e ressonância magnética, têm sido usados há muitos anos para determinar o processo distrófico em distrofias musculares. No entanto, o conhecimento a respeito da arquitetura muscular em crianças com distrofia muscular de Duchenne (DMD) em estágio inicial, com diferentes níveis funcionais, é limitado.

**Objetivo:**

Explorar o efeito do nível funcional nas propriedades arquitetônicas do músculo em crianças com DMD em estágio inicial e a diferença entre DMD e seus pares em desenvolvimento típico (DT).

**Métodos:**

Trinta crianças com DMD (15 Grau 1 e 15 Grau 2 de acordo com a Escala de Vignos) e cinco colegas DT foram incluídos. A ultrassonografia foi usada para medir a espessura muscular (EM), o comprimento do fascículo (FL) e o ângulo de penetração (PA) dos músculos vasto lateral (VL) e gastrocnêmio medial (MG) bilateralmente.

**Resultados:**

Os valores de EM e FL para VL e os valores de EM, FL e PA para músculos MG foram maiores em crianças com DMD em comparação com os de seus pares DT (p<0,05). O FL do VL e o EM e o FL dos músculos GM de crianças com DMD Grau 2 foram maiores do que aqueles de crianças com DMD Grau 1 (p<0,05).

**Conclusões:**

TM e FL estão aumentados em crianças com DMD em comparação com seus pares DT. Além disso, a arquitetura muscular parece ser afetada mesmo nos estágios iniciais da doença.

## INTRODUCTION

Duchenne muscular dystrophy (DMD) is one of the most common X-linked recessively inherited neuromuscular diseases, with an incidence of 1/3500-5000 live male births^1^ ^,^ ^2^. It results from mutations in the gene encoding dystrophin, leading to its absence^3^. The clinical scenario is primarily characterized by proximal muscle weakness, which in turn causes difficulty in standing from the supine position, frequent falls, and motor delays. Although functional performance is maintained until the age of 6 years, affected boys often lose ambulation by 12 years of age^4^. Eventually, death occurs due to cardiac and pulmonary complications within the 20s^5^.

Molecular tests and muscle imaging methods have been used for many years to diagnose and manage different neuromuscular diseases^6^. Muscle imaging methods such as magnetic resonance imaging (MRI) and ultrasound (US) are generally used in the evaluation of the dystrophic process in DMD. Although the above techniques are validated, painless, and radiation-fee methods to assess muscle pathologies^7^, US has certain advantages over MRI, such as being more child-friendly, cheaper, more convenient, and more cost-effective^8^ ^,^ ^9^. Of note, ultrasonographic data on muscle architecture play an important role in the evaluation of motor function in relevant cases^10^.

Due to the progressive nature of the disease, functional levels of children deteriorate with age. Many studies evaluating parameters such as balance, muscle strength, and pulmonary function in children found that children with low functional status had worse outcomes^11^ ^,^ ^12^ ^,^ ^13^. Additionally, it was shown that muscle echo-intensity was associated with disease severity and longitudinal changes of the dystrophic process in DMD^14^ ^,^ ^15^. Only one study has evaluated muscle architecture in adult DMD patients^16^, and there is no data in children at early stages with different functional levels.

Accordingly, the primary aim of this study was to investigate the effect of functional level on lower limb muscle architecture in children with early stage DMD. We also compared the ultrasonographic parameters of muscle architecture between DMD vs. typically developing (TD) peers.

## METHODS

### Participants

This was a cross-sectional observational study with three groups, conducted between May 2019 and March 2020 in a tertiary care university hospital. The study protocol was approved by the Clinical Research Ethics Board (Protocol Number: KA-19022). Informed consent form was obtained from the children and their families and all the procedures were performed in accordance with the Declaration of Helsinki.

Children aged 5 to 10 years were included in the study if the diagnosis of DMD was confirmed by genetic analysis, the functional level was of Grade 1 and 2 according to Vignos Scale^17^, had no comorbidities, were on corticosteroid treatment for at least for 6 months, and if they were able to cooperate with the study instructions. TD children aged 5 to 10 years were enrolled if they did not have any musculoskeletal, cognitive, neurological, or cardiopulmonary diseases. Children were excluded if they had undergone any surgery or suffered a lower limb injury.

### Assessments

Demographic characteristics (age and body mass index) were recorded before the evaluations. Functional level, muscle strength, and motor performance evaluations were consecutively performed by a physiotherapist (NB) with six years of experience in rehabilitation of pediatric neuromuscular disorders. There was a 10-minute interval between tests, which were completed within one hour.

Functional level of children with DMD was assessed using the Vignos Scale^17^, which has high intraclass correlation^18^. The scale has 10 grades, with Grade 1 denoting the best functional level and Grade 10 the worst. More specifically, children who are able to walk and climb stairs without assistance are categorized as Grade 1, while children who can walk and climb stairs with the aid of handrail are categorized as Grade 2.

Muscle strength evaluation was done for the knee extensors and ankle plantar flexors using a hand-held dynamometer (Commander, Jtech Medical Industries, Main Midvale, UT) at a standardized test position. For knee extensors, children were assessed in the sitting position with the hip and knee flexed at 90° and the dynamometer placed at ankle joint. Ankle plantar flexors were tested in the supine position with the hip in neutral and the dynamometer placed around the metatarsals^19^. Three maximal voluntary isometric contractions of both legs were performed and the mean values of the force used in the three repetitions were recorded bilaterally. Test-retest reliability of quantitative muscle testing in DMD and TD children was 0.83-0.99 and 0.74-0.99, respectively^20^.

Motor performance was assessed using timed tasks items that included rising from the floor, the 10-meter walk test, ascending/descending four stair steps and the six-minute walk test (6MWT)^21^. The completion time of the items except the 6MWT was recorded in seconds. The 6MWT consists of fast walking in an indoor a course divided by a straight line 25 m long with cones placed at the beginning and end of the line^22^. The total distance covered in the six minutes is recorded.

**Figure 1.**
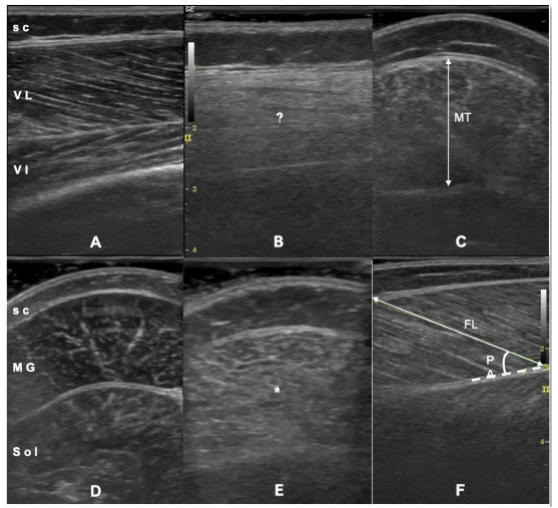
Ultrasonographic measurements of the vastus lateralis and medial gastrocnemius muscles. Longitudinal imaging for vastus lateralis in a typically developing child (A) and a Grade 2 Duchenne muscular dystrophy child (B). Note the loss of clarity (?) for visualization of the pennate structure (B). Axial imaging for muscle thickness measurement of vastus lateralis (C). Axial imaging for medial gastrocnemius in a typically developing child (D) and a Grade 1 Duchenne muscular dystrophy child (E). Note the increased echogenicity (*) of the muscle due to fibroadipose infiltration (E). Longitudinal imaging for fascicle length and pennation angle measurements of medial gastrocnemius (F).

Ultrasonographic evaluations were performed by a single physiatrist (LÖ) with 20 years of experience in musculoskeletal US using a 5-12 MHz linear probe (Logiq P5, GE Medical Systems, Wisconsin, USA). For the measurements of the vastus lateralis (VL), the children were in the supine position, with the knees in extension and the ankles in resting position. For measurement of the medial gastrocnemius (MG), they were lying in prone position with the legs extended and ankles at resting position, hanging from the side of the examination bed. Minimal pressure was applied with probe on the thickest sides of the both muscles to avoid muscle compression. For longitudinal imaging (Figurse 1A, 1B and 1F), the pennation angle (PA) was measured as the angle between the muscle fascicles and the deep aponeurosis, and the fascicle length (FL) was measured as the distance of fascicle path between the two aponeuroses. For axial imaging (Figure 1C-1E), the distance between deep and superficial aponeuroses was measured as muscle thickness (MT)^23^ ^,^ ^24^.

sc: subcutaneous fat; VL: vastus lateralis; VI: vastus intermedius muscle; MT: muscle thickness; MG: medial gastrocnemius; Sol: soleus muscle; FL: fascicle length; PA: pennation angle.

### Statistical analysis

The IBM Statistical Package for the Social Sciences (SPSS), version 20.0, was used for statistical analyses. Kolmogorov-Smirnov test was used to determine normal distribution. Demographic and ultrasonographic parameters were compatible with parametric conditions while muscle strength and motor performance assessments with non-parametric conditions. Descriptive statistics for quantitative data are given as mean±standard deviation (SD) or as median and interquartile range (25^th^ and 75^th^ percentiles). Mann-Whitney U or independent sample t-test was used to compare children with DMD with TD peers, as appropriate. Statistical significance was accepted when p<0.05. Spearman coefficients were used for correlation analyses and classified as very high (0.90-1.0), high (0.70-0.90), moderate (0.50-0.70), low (0.30-0.49), and negligible (0-0.30)^25^.

## RESULTS

Thirty-one children with DMD and five TD peers were enrolled. Only one child with DMD was excluded because of poor cooperation. Overall, data of 35 children (70 lower limbs) were included in the analyses.

Mean age of children with DMD and that of TD peers was 8.83±1.55 and 8.10±1.12 years, respectively. Mean body mass index of children with DMD was 17.06±2.05 kg/m^2^ and that of TD peers was 16.55±0.78 kg/m^2^. Demographic characteristics were similar between the two groups (all p>0.05). In the DMD group, 15 children had functional level of Grade 1 and 15 had Grade 2.

Data regarding muscle strength and motor performance are shown in Table 1. Muscles strength (except for the dominant side ankle plantar flexors) and motor performance were worse in children with DMD compared with TD children (all p<0.05). DMD children with Grade 1 functional level had higher knee extensor strength and better motor performance compared with those with Grade 2 (all p<0.05).

**Table 1.** Strength and functional assessment results (median, 25^th^-75^th^ range).

	DMD		p-value^c^	TD peers (n=5)	p-value^d^
	Grade 1 (n=15)	Grade 2 (n=15)			
Strength (D)^a^	15.73 (13.80-21.00)	9.30 (6.30-10.80)	<0.001*	32.50 (31.86-45.75)	0.001*
Strength (ND)^a^	16.50 (13.20-19.50)	8.80 (6.86-12.00)	<0.001*	32.00 (28.79-34.65)	<0.001*
Strength (D)^b^	26.33 (24.23-29.97)	26.10 (22.80-28.80)	0.9	32.00 (24.30-37.40)	0.1
Strength (ND)^b^	23.00 (21.27-27.37)	26.50 (21.00-30.50)	0.5	32.00 (28.79-34.65)	0.007*
Rising from floor (sec)	4.75 (3.01-5.25)	13.75 (9.00-33.92)	<0.001*	2.09 (1.10-2.30)	<0.001*
10-m walk (sec)	7.56 (5.88-8.19)	8.45 (7.43-11.42)	0.01*	5.95 (4.97-6.22)	0.005*
Ascending 4 steps (sec)	2.39 (2.08-2.92)	8.83 (3.66-11.44)	<0.001*	1.76 (1.05-2.17)	0.001*
Descending 4 steps (sec)	2.15 (1.83-2.75)	4.40 (2.78-7.76)	0.007*	1.63 (1.25-1.84)	0.003*
6-min walk test (m)	443.00 (410.00-473.00)	350.00 (300.00-390.00)	<0.001*	580.00 (538.00-595.50)	<0.001*

n: number of participants; DMD: Duchenne muscular dystrophy; D: dominant; ND: non-dominant; TD: typically developing; ^a^Muscle strength of knee extensors; ^b^Muscle strength of ankle plantar flexors; *p<0.05; ^c^Grade 1 vs. Grade 2; ^d^DMD vs. TD peers.

Ultrasonographic measurements are shown in Table 2. Muscle architecture parameters regarding VL could not be obtained from 3 children (20%) with Grade 1 and 6 children (40%) with Grade 2 DMD because of excessive muscle echogenicity. FL of the VL muscle and MT and FL of the MG muscle were lower in children with DMD of Grade 1 compared to Grade 2 (all p<0.05). MT and VL values of the VL muscle and MT, FL, and PA values of the MG muscle were higher in children with DMD compared to TD peers (all p<0.05).

**Table 2.** Ultrasonographic measurements of the subjects’ lower limbs (mean±SD).

VL muscle	DMD			TD peers (n=10)	p-value^b^
	Grade 1 (n=24)	Grade 2 (n=18)	p-value^a^		
MT (cm)	2.03±0.33	2.26±0.47	0.07	1.90±0.72	0.002*
FL (cm)	6.45±1.32	7.93±0.98	<0.001*	6.04±0.84	0.006*
PA (degree)	18.78±3.80	19.41±3.52	0.5	19.09±2.88	0.9
**GM muscle**	**DMD**			**TD peers (n=10)**	**p-value^b^**
	**Grade 1 (n=30)**	**Grade 2 (n=30)**	**p-value^a^**		
MT (cm)	1.83±0.32	2.18±0.48	0.002*	1.36±0.14	<0.001*
FL (cm)	3.47±0.67	4.27±0.99	0.001*	2.97±0.36	<0.001*
PA (degree)	30.87±4.20	30.97±5.33	0.9	25.25±2.67	<0.001*

SD: standard deviation; n: number of limbs; DMD: Duchenne muscular dystrophy; MG: medial gastrocnemius; VL: vastus lateralis; MT: muscle thickness, FL: fascicle length; PA: pennation angle; TD: typically developing. *p<0.05. ^a^Grade 1 vs. Grade 2, ^b^DMD vs. TD peers.

Correlations among ultrasonographic measurements, muscle strength, and motor performance are shown in Table 3. Concerning the VL muscle, FL was negatively correlated with muscle strength and 6MWT and positively correlated with time for rising from the floor and ascending four stair steps (all p<0.05). Concerning the MG muscle, FL was negatively correlated with the 6MWT and positively correlated with time for rising from the floor and ascending four stair steps. Low correlations were found between VL MT and the 10-meter walk test and between MG MT and time for rising from the floor and ascending four stair steps (all p<0.05).

**Table 3.** Correlations between ultrasonographic and functional assessment results in children with Duchenne muscular dystrophy.

VL (n=21)	MT (D)	MT (ND)	FL (D)	FL (ND)	PA (D)	PA (ND)
Strength (D)^a^	-0.13	-0.22	-0.38	-0.46*	-0.08	0.13
Strength (ND)^a^	-0.11	-0.23	-0.40	-0.44*	-0.07	0.07
Rising from the floor	0.30	0.24	0.62*	0.47*	0.24	0.17
Ten-meter walk test	0.44*	0.26	0.29	0.13	0.11	0.29
Ascending four steps	0.29	0.27	0.43*	0.37	0.33	0.10
Descending four steps	0.13	0.08	0.07	-0.19	0.06	0.13
Six-minute walk test	-0.23	-0.24	-0.45*	-0.27	-0.10	-0.26
**MG (n=30)**	**MT (D)**	**MT (ND)**	**FL (D)**	**FL (ND)**	**PA (D)**	**PA (ND)**
Strength (D)^b^	0.01	0.03	0.23	0.23	-0.09	-0.05
Strength (ND)^b^	-0.05	-0.03	0.34	0.31	-0.24	-0.19
Rising from the floor	0.42*	0.28	0.46*	0.52*	-0.02	-0.05
Ten-meter walk test	0.07	0.01	0.24	0.25	0.00	0.07
Ascending four steps	0.37*	0.28	0.41*	0.47*	0.00	0.05
Descending four steps	0.27	0.23	0.16	0.08	0.11	0.20
Six-minute walk test	-0.30	-0.17	-0.39*	-0.36	0.07	-0.06

n: number of participants; D: dominant; ND: non-dominant; DMD: Duchenne muscular dystrophy; MG: medial gastrocnemius; VL: vastus lateralis; MT: muscle thickness; FL: fascicle length; PA: pennation angle. Muscle strength of knee extensors^a^ and ankle plantar flexors^b^ were presented. *p<0.05.

## DISCUSSION

This study explored the effect of functional level on lower limb muscle architecture in children with early stage DMD and compared it with that of TD peers. To our best knowledge, this is the first US study involving children with different functional levels demonstrating that the architecture of the VL and MG muscles is affected even at early stages of the disease, and the changes might also be related with functional deterioration.

Absence of dystrophin in DMD makes the sarcolemma fragile and easily damaged by stresses that develop during muscle contractions^3^. Furthermore, sarcolemmal damage is accompanied by muscle fiber necrosis and inflammation, and muscle tissue is replaced by fat and connective tissue^3^ ^,^ ^16^. Indeed, muscle architectural parameters of VL could not be obtained in 20% of children with Grade 1 and in 40% of children with Grade 2 DMD in our study. Gradually, this replacement results in ‘pseudohypertrophy’ i.e., excessive increase in muscle size. Of note, the muscle groups in which pseudohypertrophy occurs in children with DMD are the quadriceps and plantar flexors^26^ ^,^ ^27^. In this sense, and in agreement with the literature, the fact that MT was higher in our DMD children could be due to pseudohypertrophy. Likewise, the reason why Grade 2 DMD children - who had worse functional level - also had a higher MT for MG than Grade 1 children could be the inflammatory process mentioned earlier. Although the MT of VL was higher in Grade 2 DMD than in Grade 1 DMD, the lack of statistical significance might be due to missing/small data.

Concerning muscle architecture, Lovering et al.^28^ reported that the increased muscle volume due to pseudohypertrophy may correlate with higher PA and maintained FL in Mdx mice. On the other hand, it was found that PA and FL were not different in adult DMD patients compared with healthy controls^16^. Our results have shown that the FL of VL and the PA and FL of MG were higher in children with DMD and that the FL of both muscles increased with worsening functional level. These inconsistencies may be due to heterogeneous patient populations with different demographic and disease characteristics. Nonetheless, muscle architecture seems to be impaired in children with DMD starting from early stages.

Outcome measures from muscle strength and motor performance are often taken into consideration in the prognosis of DMD. Studies have found that these two measures are lower in children with DMD even at the early stage^13^ ^,^ ^29^. As expected, our findings revealed that the knee extensor and ankle plantar flexor (only of the non-dominant leg) strength and motor performance were better in TD children (vs. DMD). Between DMD groups, knee extensor strength and motor performance were worse in Grade 2 than in Grade1. We attribute the indifference in ankle plantar flexor strength between groups to the fact that distal muscle groups were less affected at the early stages of disease.

MT negatively correlated with motor performance. In other words, increased MT was associated with longer duration of timed performance tests. Similarly, one MRI study in children with DMD reported worse timed performance test results despite increased cross sectional area of triceps surae muscle^26^. These findings reaffirm the fact that the “pathological” hypertrophy (i.e. pseudohypertrophy) caused by inflammatory fat infiltration is different than the normal physiological development of TD peers. Additionally, similar to MT, increased FL was correlated with weaker muscle strength and worse motor performance. Thus, low or no correlations between muscle architecture and physical assessment parameters may be explained by additional compensatory movements of children with DMD during functional tasks^30^.

The major limitation of this study was its small sample size (especially for the TD group). However, the uncertainty related with the current COVID-19 pandemic complicated subject enrollment in several ways and the study had to be terminated earlier than originally planned.

To summarize, in the light of our study results, two main conclusions can be drawn. First, MT is increased and functional performance is decreased in children with DMD and second, muscle architecture is affected even at the early stages of the disease. Further studies that include children with a diagnosis of other neuromuscular diseases and peers matched for physical activity level in addition to children in the advanced stages of DMD are needed. Also, the effect of therapeutic approaches on muscle architectural properties in DMD should be investigated in future clinical trials.

## References

[1] Emery AE (1991). Population frequencies of inherited neuromuscular diseases-a world survey. Neuromuscul Disord.

[2] Mendell JR, Shilling C, Leslie ND, Flanigan KM, al-Dahhak R, Gastier-Foster J (2012). Evidence-based path to newborn screening for Duchenne muscular dystrophy. Ann Neurol.

[3] Hoffman EP, Brown RH, Kunkel LM (1987). Dystrophin: the protein product of the Duchenne muscular dystrophy locus. Cell.

[4] Emery AE (2002). The muscular dystrophies. Lancet.

[5] Manzur AY, Kinali M, Muntoni F (2008). Update on the management of Duchenne muscular dystrophy. Arch Dis Child.

[6] Simon NG, Noto Y-i, Zaidman CM (2016). Skeletal muscle imaging in neuromuscular disease. J Clin Neurosci.

[7] Kim HK, Merrow AC, Shiraj S, Wong BL, Horn PS, Laor T (2013). Analysis of fatty infiltration and inflammation of the pelvic and thigh muscles in boys with Duchenne muscular dystrophy (DMD): grading of disease involvement on MR imaging and correlation with clinical assessments. Pediatr Radiol.

[8] Scholten R, Pillen S, Verrips A, Zwarts MJ (2003). Quantitative ultrasonography of skeletal muscles in children: normal values. Muscle Nerve.

[9] Pillen S, Verrips A, van Alfen N, Arts IMP, Sie LTL, Zwarts MJ (2007). Quantitative skeletal muscle ultrasound: diagnostic value in childhood neuromuscular disease. Neuromuscul Disord.

[10] Blazevich AJ, Cannavan D, Coleman DR, Coleman DR, Horne S (2007). Influence of concentric and eccentric resistance training on architectural adaptation in human quadriceps muscles. J Appl Physiol (1985).

[11] Phillips MF, Quinlivan RC, Edwards RH, Calverley PM (2001). Changes in spirometry over time as a prognostic marker in patients with Duchenne muscular dystrophy. Am J Respir Crit Care Med.

[12] Alkan H, Mutlu A, Fırat T, Bulut N, Karaduman AA, Yılmaz OT (2017). Effects of functional level on balance in children with Duchenne Muscular Dystrophy. Eur J Paediatr Neurol.

[13] Nunes MF, Hukuda ME, Favero FM, Oliveira AB, Voos MC, Caromano FA (2016). Relationship between muscle strength and motor function in Duchenne muscular dystrophy. Arq Neuro-Psiquiatr.

[14] Jansen M, van Alfen N, van der Sanden MWN, van Dijk JP, Pillen S, de Groot IJM (2012). Quantitative muscle ultrasound is a promising longitudinal follow-up tool in Duchenne muscular dystrophy. Neuromuscul Disord.

[15] Zaidman CM, Wu JS, Kapur K, Pasternak A, Madabusi L, Yim S (2017). Quantitative muscle ultrasound detects disease progression in Duchenne muscular dystrophy. Ann Neurol.

[16] Morse C, Smith J, Denny A, Tweedale J, Searle ND (2015). Gastrocnemius medialis muscle architecture and physiological cross sectional area in adult males with Duchenne muscular dystrophy. J Musculoskelet Neuronal Interact.

[17] Vignos P, Archibald K (1960). Maintenance of ambulation in childhood muscular dystrophy. J Chronic Dis.

[18] Florence JM, Pandya S, King WM, Robison JD, Signore LC, Wentzell M (1984). Clinical trials in Duchenne dystrophy: standardization and reliability of evaluation procedures. Phys Ther.

[19] Hogrel J-Y, Payan CA, Ollivier G, Tanant V, Attarian S, Couillandre A (2007). Development of a French isometric strength normative database for adults using quantitative muscle testing. Arch Phys Med Rehabil.

[20] Stuberg WA, Metcalf W (1988). Reliability of quantitative muscle testing in healthy children and in children with Duchenne muscular dystrophy using a hand-held dynamometer. Phys Ther.

[21] Mazzone E, Martinelli D, Berardinelli A, Messina S, D’Amico A, Vasco G (2010). North Star Ambulatory Assessment, 6-minute walk test and timed items in ambulant boys with Duchenne muscular dystrophy. Neuromuscul Disord.

[22] ATS Committee on Proficiency Standards for Clinical Pulmonary Function Laboratories (2002). ATS statement: guidelines for the six-minute walk test. Am J Respir Crit Care Med.

[23] Kaya A, Kara M, Tiftik T, Tezcan ME, Ozel S, Ersöz M (2013). Ultrasonographic evaluation of the muscle architecture in patients with systemic lupus erythematosus. Clin Rheumatol.

[24] Karamanidis K, Arampatzis A (2006). Mechanical and morphological properties of human quadriceps femoris and triceps surae muscle-tendon unit in relation to aging and running. J Biomech.

[25] Mukaka MM (2012). A guide to appropriate use of correlation coefficient in medical research. Malawi Med J.

[26] Mathur S, Lott DJ, Senesac C, Germain SA, Vohra RS, Sweeney HL (2010). Age-related differences in lower-limb muscle cross-sectional area and torque production in boys with Duchenne muscular dystrophy. Arch Phys Med Rehabil.

[27] Jones D, Round J, Edwards R, Grindwood SR, Tofts PS (1983). Size and composition of the calf and quadriceps muscles in Duchenne muscular dystrophy: a tomographic and histochemical study. J Neurol Sci.

[28] Lovering RM, Shah SB, Pratt SJ, Gong W, Chen Y (2013). Architecture of healthy and dystrophic muscles detected by optical coherence tomography. Muscle Nerve.

[29] Beenakker EA, Maurits NM, Fock JM, Brouwer OF, van der Hoeven JH (2005). Functional ability and muscle force in healthy children and ambulant Duchenne muscular dystrophy patients. Eur J Paediatr Neurol.

[30] Martini J, Voos MC, Hukuda ME, Dutra de Resende MB, Caromano FA (2014). Compensatory movements during functional activities in ambulatory children with Duchenne muscular dystrophy. Arq Neuro-Psiquiatr.

